# Erythropoietin in Lupus: Unanticipated Immune Modulating Effects of a Kidney Hormone

**DOI:** 10.3389/fimmu.2021.639370

**Published:** 2021-03-16

**Authors:** Meghana Eswarappa, Chiara Cantarelli, Paolo Cravedi

**Affiliations:** ^1^Department of Medicine, Icahn School of Medicine at Mount Sinai, New York, NY, United States; ^2^UO Nefrologia, Azienda Ospedaliero-Universitaria di Parma, Parma, Italy

**Keywords:** erythropoietin, SLE, immunology, lupus, T cell, Treg

## Abstract

Systemic lupus erythematosus (SLE) is a multiorgan autoimmune disease with variable clinical presentation, typically characterized by a relapsing-remitting course. SLE has a multifactorial pathogenesis including genetic, environmental, and hormonal factors that lead to loss of tolerance against self-antigens and autoantibody production. Mortality in SLE patients remains significantly higher than in the general population, in part because of the limited efficacy of available treatments and the associated toxicities. Therefore, novel targeted therapies are urgently needed to improve the outcomes of affected individuals. Erythropoietin (EPO), a kidney-produced hormone that promotes red blood cell production in response to hypoxia, has lately been shown to also possess non-erythropoietic properties, including immunomodulatory effects. In various models of autoimmune diseases, EPO limits cell apoptosis and favors cell clearance, while reducing proinflammatory cytokines and promoting the induction of regulatory T cells. Notably, EPO has been shown to reduce autoimmune response and decrease disease severity in mouse models of SLE. Herein, we review EPO's non-erythropoietic effects, with a special focus on immune modulating effects in SLE and its potential clinical utility.

## Introduction

Systemic lupus erythematosus (SLE) is a complex autoimmune disorder with multiorgan involvement. Interactions amongst genetic, hormonal and environmental factors lead to immune dysregulation and loss of tolerance to self-antigens, with consequent autoantibody production, inflammation, and tissue damage (1). SLE is characterized by a relapsing-remitting course with a wide spectrum of clinical presentations, including—but not limited to—cutaneous, articular, hematologic, pulmonary, neurological and renal complications. In particular, the prevalence of neurological manifestations, both of the central and peripheral nervous system, ranges between 14 and 95% and is associated with worse outcomes and higher mortality rates (2). The pathogenesis of neural disease in SLE remains unclear, but it likely involves a direct role of autoantibodies, inflammatory cytokines and chemokines, and brain blood barrier dysfunction (3). Renal disease affects between 28 and 74% of SLE patients and is also associated with increased mortality (4–6). Despite treatment, a substantial percentage of SLE patients still develops end stage kidney disease (ESKD) and disease may recur after kidney transplantation (7, 8).

Available therapeutic options for SLE have limited efficacy and are burdened by significant toxicities. Therefore, new, hypothesis-driven therapies are needed to improve the outcomes of individuals with SLE.

### SLE Pathogenesis

Our understanding of SLE pathogenesis is still incomplete, but the following mechanisms are thought to play a major role. Defective clearance of debris from apoptotic cells exposes nuclear antigens, which initially triggers an innate inflammatory response via activation of toll-like receptors (TLR) and then bolsters T cell and B cell responses against autoantigens (9, 10). B cells present autoantigens to T cells, produce autoantibodies, and promote local inflammation. The autoantibodies bind to self-antigens and form immune complexes in various organs, further fueling the autoinflammatory response through the activation of complement and the recruitment of FcyR- and TLR-expressing innate immune cells. In turn, these cells release proinflammatory cytokines and chemokines, sustaining leukocyte infiltration and activation and formation of lymphoid aggregates, leading to organ damage (10, 11).

#### Dendritic Cells and Macrophages

Multiple abnormalities in dendritic cells (DCs) have been identified in SLE patients. In particular, plasmacytoid DCs (pDCs), responsible for secretion of high levels of type I interferon (IFN) via TLR7 and TLR9 stimulation, are increased in patients with SLE (12). Sustained production of type I IFN by pDCs in response to immune complexes represents a hallmark of SLE (13). Importantly, massive pDCs infiltrates are found in renal and skin lesions of SLE patients (14). This, together with the observation that ablation of these cells in lupus-prone mice reduces autoantibody production and lupus nephritis disease severity (15), supports the role of pDCs in the pathogenesis of SLE. Increase in pDCs is paralleled by a decline in conventional DCs (cDCs) in peripheral blood of SLE patients (16). These DCs are involved in maintaining self-tolerance and their reduction leads to an imbalance in DC subsets that favors a proinflammatory environment (17, 18).

Impaired clearance of apoptotic cell debris is a central pathogenic mechanism in the development of SLE. Defective clearance of apoptotic cell debris promotes release of autoantigens and autoreactive B cell stimulation, which leads to loss of tolerance and generation of autoantibodies. Consequent immune complex formation and deposition results in organ damage (19). As macrophages are a key cell subset in the clearance of apoptotic debris, it is not surprising that defective macrophage activity contributes to the pathogenesis and correlates with disease severity (20). Macrophage infiltrates in the kidney represent a strong prognostic biomarker for progression of lupus nephritis and correlates with the disease activity index (21).

#### B Cells

The role of B cells in the development of SLE has recently raised interest, not only for their ability to produce autoantibodies that lead to organ damage, but also for complex interactions with other cell types. Immune phenotypic studies showed abnormalities in the proportion of different B cell subsets in SLE individuals. In particular, B cell lymphopenia with reduced numbers of naïve B cells and an increase in circulating class-switched memory B cells, plasma blasts and plasma cells is observed and correlates with disease activity (22).

Under the influence of genetic susceptibility and environmental factors, B cells in SLE patients show increased activation, as documented by active B cell receptor (BCR) signaling with increased phosphorylation of PI3K and AKT-1 and abnormal phosphatase activity (23), increased production of cytokines IL-6 and IL-10, constitutive expression of costimulatory molecules that affect T cell function and antigen presenting cells (APCs) (24), and loss of tolerance.

#### T Cells

Murine and human data converge to indicate that SLE is associated with defective and/or decreased numbers of regulatory T cells (Treg), which normally act to control conventional T cells (Tconv) and promote self-tolerance (25). Tconv in SLE individuals also display abnormalities that are likely the result of primary defects and the consequence of the proinflammatory environment. T cell abnormalities include altered activation signaling pathways, increased expression of pro-migratory markers, and upregulation of co-stimulatory CD40 ligand, contributing to B-cell activation. T cells from SLE patients also show an altered cytokine profile, including decreased transforming growth factor beta (TGF-β) and IL-2, and increased IL-6 and IL-17 expression, which may contribute to the imbalance in T cell subsets (26, 27).

In particular, increased IL-17 and decreased IL-2 levels account for the higher Th17/Th1 ratio reported in SLE compared to healthy controls (28). Altered IL-2 production is also associated with Treg dysfunction and further promotes expression of IL-17, with a decreased Treg/Th17 ratio, which is detectable not only during flares, but also when the disease is in remission (29, 30). SLE patients show increased Th17 cells in peripheral blood and in kidney and skin lesion infiltrates, as well (31). SLE patients also display an imbalanced Th1/Th2 ratio, which is thought to play a major role in disease pathogenesis. Plasma levels of IL-10, a main driver of Th2 differentiation, are significantly increased and correlate with disease activity (32).

### EPO and EPO Receptors

Erythropoietin is a glycoprotein initially discovered for its role in stimulating red blood cell production. More recently, evidence has accumulated indicating that EPO also displays non-erythropoietic properties. Interstitial fibroblasts in the kidney produce a basal level of EPO which binds to receptors on erythroid progenitor cells in the bone marrow to maintain a steady red blood cell mass (33, 34). Tissue hypoxia increases EPO production by stabilizing the Hypoxia Inducible Factor (HIF) transcriptional complex and activating EPO gene transcription (35).

Studies have identified two distinct EPO receptors. One is a homodimer receptor consisting of two EPO receptor (EPOR) monomers. Activation of this homodimer on erythroid progenitor cells triggers downstream signaling via JAK2 and subsequently STAT5, MAPK and PI3K pathways (36) which maintains erythropoiesis. The other receptor is a heterodimer, consisting of an EPOR monomer subunit and the β-common receptor CD131. EPOR-CD131 requires a higher concentration of EPO for activation and has been implicated in the non-erythropoietic, “tissue-protective” effects of EPO, due to its downstream effects that mediate suppression of proinflammatory cytokines and inhibition of apoptosis (37–39).

### EPO Derivatives

Current FDA-approved indications for EPO include treatment of anemia associated with chronic kidney disease (CKD) or chemotherapy (40, 41). The increased risk of thrombosis and stroke associated with EPO administration (39, 40, 42–44) prompted researchers to design asialoerythropoietin, a desialytated version of recombinant EPO notable for its shorter half-life which allowed for its neuroprotective effects with limited effects on erythrocyte mass (45).

An alternative approach was to develop molecules that selectively bind the EPOR-CD131 heterodimer and are therefore devoid of erythrogenic effects associated with the activation of the EPOR homodimer. This gave rise to carbamylated EPO (produced by carbamylation of lysine residues) and ARA290 (an 11-amino acid peptide that mimics EPO's helix B region), which have also been shown to maintain EPO's tissue-protective but not hematopoietic effects (39, 46, 47).

### EPO's Non-erythropoietic Effects

Over the last few decades, many non-erythropoietic effects of EPO have been identified in multiple organs. In the nervous system, EPOR expression has been detected in neurons, astrocytes, oligodendrocytes, microglia, and endothelial cells. Importantly, animal studies have shown that EPO has neuroprotective effects via neurogenesis, angiogenesis and anti-apoptotic, anti-oxidative, and anti-inflammatory mechanisms (48). Although one clinical trial of EPO in the treatment of acute ischemic stroke found that EPO administration within 6 h of symptoms was associated with increased mortality (49), another trial suggested that EPO administration post-acute ischemic stroke in non-tPA (tissue plasminogen activator) candidates was associated with improved long-term neurological outcomes (50). EPO showed promising neuroprotective effects also in animal models of autoimmune optic neuritis (51), setting the basis for a clinical trial in humans (NCT01962571) (52). Further ophthalmological effects have been noted, including protection against retinal degeneration (53–55).

In the cardiovascular system, both endothelial cells and cardiomyocytes express EPORs. In experimental studies, EPO protects against cardiac ischemic injury by decreasing apoptosis and inflammation, and by promoting neovascularization (56). However, clinical trials of EPO administration after myocardial infarction (MI) have reported mixed results (57, 58) and a meta-analysis on 1,336 patients showed no improvement in infarct size, left ventricular function, or mortality when EPO was administered in patients undergoing percutaneous coronary revascularization post-MI (59).

EPOR has also been localized in renal tubular and mesangial cells (60). In animal models of kidney injury, such as ischemic-reperfusion injury, erythropoiesis stimulating agents (ESA), including EPO derivatives, have improved disease severity via anti-apoptotic effects (61, 62). However, this beneficial effect has largely not been reflected in clinical trials. A meta-analysis of clinical trials found no clear benefit to ESAs in the development of acute kidney injury primarily following cardiac surgery, in renal transplant outcomes, or in CKD progression after anemia correction (63).

Therefore, tissue-protective effects of EPO have been largely demonstrated in numerous models of organ injury, but their clinical translation has provided inconsistent results, possibly as consequence of suboptimal dosing and timing. Whether selective activation of non-erythropoietic EPOR would improve safety/efficacy profile of EPO is worth investigating.

### EPO's Anti-oxidative and Anti-apoptotic Effects

Oxidative stress contributes to tissue damage in the brain, kidney, heart and other organs. The discovery that EPO has direct and indirect anti-oxidative effects supports its use as a tissue-protective molecule. Anti-oxidative properties of EPO are in part independent from its role in countering apoptosis. EPO increases gene expression of Heme-Oxygenase 1 and other anti-oxidative enzymes, like superoxide dismutase, catalase, and glutathione peroxidase, directly on the cells, without the involvement of erythroid cell progenitors (64).

Several studies in different disease models and tissues identified the JAK2-STAT-Bcl2 pathway as one of the main anti-apoptotic mechanisms of EPO, through the induction of anti-apoptotic molecules, Bcl-2 and Bcl-Xl, and the inhibition of pro-apoptotic molecules, Bax and Bak (38). In erythroid cells, EPO-EPOR interactions prevent apoptosis through STAT5 signaling (65). In a murine model of acute encephalopathy due to cerebral malaria, EPO was associated with a dose dependent improvement in survival, together with a significantly reduced number of apoptotic cells (66). In a middle-cerebral artery model of ischemic injury in rats, EPO rescued neurons from apoptosis in a time-dependent manner, through activation of extracellular signal-regulated kinases and PI3K (67). Furthermore, EPO has been noted to exert direct protective effects on pancreatic β islet cells in diabetes mouse models (68), and, in neonatal porcine islet cells, EPO's anti-apoptotic effect occurs through upregulation of Bcl-2 mRNA and downregulation of Bax and caspase-3 mRNA (69).

### EPO's Immunomodulatory Effects

#### Innate Immunity

Erythropoietin's immunomodulatory activity has been demonstrated in both innate and adaptive immune pathways (70) (Table 1). In animal models of various autoimmune diseases, EPO reduced disease severity and was associated with decreased levels of proinflammatory cytokines. In a rat model of experimental autoimmune encephalomyelitis, EPO administration resulted in a dose-dependent delay in disease onset and decreased disease severity, as well as decreased inflammatory cells including macrophages, microglia, dendritic cells and monocytes. In this model, EPO also delayed the rise in tumor necrosis factor (TNF) levels and decreased the peak of IL-6 levels in the spinal cord (72). Nairz et al. showed that EPO inhibits NF-kB and subsequently reduces expression of proinflammatory genes (Nos2, TNF-α, and IL-6) in murine macrophages. Consistently, EPO administration reduced disease severity in experimental mouse models of autoimmune colitis. The anti-inflammatory effects of EPO, in contrast, impaired clearance of bacterial colonies in *Salmonella typhimurium*-infected mice, reducing animal survival (73). In mice with collagen-induced arthritis, EPO significantly reduced disease severity, oxidative damage, levels of proinflammatory cytokine TNF-α and chemokines MIP-1a and MIP-2, neutrophil infiltration, and the levels of chondrocyte apoptosis (80).

**Table 1:** Role of various cell subsets in SLE pathogenesis and effects of EPO.

**Role in SLE**	**EPO effects**
**Innate Immune cells**	
**Dendritic cells**	
cDCs are reduced, favoring a proinflammatory environment. pDCs produce high amounts of type I IFN that stimulate B cell proliferation, inflammation and loss of tolerance, promoting SLE development.	- In mice with cerebral malaria, EPO inhibits DCs differentiation and their expression of CD80, CD86, and TLRs (71) - EPO reduces number of DCs in rat EAE model (72)
**Macrophages**	
Macrophages have impaired function and cell clearance ability. Kidney macrophage infiltrates correlate with disease activity	- EPO inhibits NF-kB and reduces expression of pro-inflammatory genes (Nos2, TNF-α, and IL-6) in mice (73) - EPO downregulates the expression of inflammatory cytokines by macrophages (71) - In pristane-induced lupus-like murine model, EPO increases phagocytosis of apoptotic cells by macrophages and reduces accumulation of dying cells (74)
**Adaptive Immunity**	
**Th1**	
SLE patients show altered cytokine profile, including decreased IL-2 plasma levels, which contribute to the imbalance in T cell subsets	- EPO reduces Th1 proliferation, without affecting cell survival (75) - EPO reduces Th1 in MRL/lpr mice (76) - EPO decreases Th1 in rats with EAN (77)
**Th2**	
IL-10 plasma levels, main drivers of Th2 differentiation, are increased and correlate with SLE disease activity	- EPO promotes Th2 differentiation in rat model of EAN (77) - It increases Th2 cells in MRL/lpr mice (76)
**Th17**	
Th17 are increased and promote inflammation and tissue damage. These cells are found in kidney and skin infiltrates	- EPO prevents RORC expression and Th17 induction (78) - It promotes Th17 conversion into Treg (78) - EPO reduces Th17 in MRL/lpr mice and in pristane-induced SLE in mice (76, 78)
**Treg**	
Treg are decreased or defective, contributing to a proinflammatory environment and loss of self-tolerance	- EPO promotes Treg induction through the release of active TGF-β by APCs (79) - EPO increases Treg in lymph nodes and in CNS in mice with EAE (72) - EPO increases Treg in MRL/lpr mice (76, 78) - EPO increases Treg in heart-transplanted mice (79)
**B cells**	
B cells produce autoantibodies and function as defective APCs that mediate T cells' loss of tolerance	- No direct effects of EPO on B cells have been reported.

*EPO, erythropoietin; SLE, systemic lupus erythematosus; cDCs, conventional dendritic cells; pDCs, plasmacytoid dendritic cells; IFN, interferon; TLRs, Toll-like receptors; NF-kB, nuclear factor kappa-light-chain-enhancer of activated B cells; Nos2, nitric oxide synthase 2; TNF-α, tumor necrosis factor α; EAN, experimental autoimmune neuritis; RORC, RAR-related orphan receptor C; TGF-β, transforming growth factor β; Treg, regulatory T cells; APCs, antigen presenting cells; EAE, experimental autoimmune encephalomyelitis; CNS, central nervous system*.

Both murine and human DCs express EPOR, suggesting that DCs can participate in the immunomodulatory properties of EPO. In DCs, EPO/EPOR signaling is more dependent on STAT3 than STAT5 (81). In studies of mice with cerebral malaria, EPO treatment significantly inhibited DCs differentiation and reduced expression of costimulatory markers CD80 and CD86, and TLRs (71).

Erythropoietin has also been demonstrated to play a role in macrophage clearance of apoptotic cells. The “find-me signal” sphingosine 1-phosphate released by dying cells activates EPO signaling in macrophages and, through upregulation of peroxisome proliferator activated receptor-y (PPARy), improves clearance of apoptotic cells (74). EPO-derivative ARA290 decreases expression of TNF-α and iNOS in LPS-treated macrophages and increases phagocytosis of apoptotic cells as well (82).

#### Adaptive Immunity

Both human peripheral blood T and B lymphocytes express EPOR (83), but the effects of EPO/EPOR interaction have been mainly characterized in T cells subsets.

#### Th1

Our previous experiments showed that EPO reduces Tconv proliferation in a dose-dependent manner, without affecting cell survival, and reduces Th1 polarization. These effects are mediated by the homodimeric EPO-R expressed on T cells that interferes with signaling downstream of the IL-2R β chain, required for Tconv functions (75). The result is supported by the fact that ARA290 affects proliferation of anti-CD3/anti-CD28 mAb-stimulated CD4+ T cells (75).

#### Th2

Th2 differentiation of human naïve CD4+ T cells is not affected by EPO *in vitro* (75). Conversely, *in vivo* studies in experimental autoimmune neuritis model in rats show that treatment with EPO or ARA290 promotes Th2 differentiation and, together with Th1 and Th17 reduction and Treg increase, improves the disease (77, 84).

#### Th17

Th17 cells are strongly linked to autoimmunity and have a main role in SLE pathogenesis.

*In vitro* treatment with EPO of CD4+ T cells under Th17 polarizing conditions, prevents Th17 master regulator RAR-related orphan receptor C (RORC) and Th17 gene expression and Th17 cell induction, even after exposure to high concentrations of NaCl, a potent Th17 inducer, without affecting cell survival (78). EPO-EPOR interaction also prevents serine-threonine protein kinase-1 (SGK1) phosphorylation, required for RORC activity. SGK1 phosphorylation is dependent upon p38 mitogen-activated protein kinase, which is counteracted by EPO (78). *In vitro* experiments confirmed that EPO prevents Th17 induction and promotes the conversion of Th17 into Treg (78).

#### Treg

*In vitro*, EPO promotes the release of active TGF-β from APCs. As TGF-β is the main driver of naïve CD4+ T cell conversion into Treg, EPO thus promotes Treg induction. Importantly, while EPO inhibits Tconv proliferation, it does not affect Treg function once they are formed. Indeed, EPO uncouples signaling downstream of the IL-2R β chain, which is already silenced in Treg by internal phosphatases, leaving IL-2R γ chain signaling, crucial for T cells, unaffected (79).

EPO treatment increases Treg also *in vivo* in experimental models of autoimmune encephalitis (85), SLE (76, 78) and organ transplantation (79). Importantly, the administration of EPO in doses required to correct anemia resulted in increased frequency of peripheral Treg in humans with CKD (79).

## EPO IN SLE

### Anti-EPO and Anti-EPOR Autoantibodies

Most EPO-related research in SLE has focused on the association between anemia and autoantibodies to EPO and EPOR. Autoantibodies to EPO in patients with SLE were first demonstrated by Tziuofas et al. (86). Since then, several studies have reported associations between the presence of anti-EPO antibodies and hematological (EPO or hemoglobin/hematocrit levels) and SLE-related parameters (SLE disease activity, complement levels or anti-dsDNA antibody levels) (86–88). Overall, these studies found an impaired EPO response in anemic SLE patients, suggesting that autoantibodies may act as EPO antagonists (87, 88). However, other reports indicate that anti-EPO antibodies may just interfere with serum EPO measurement rather than inhibit EPO activity (88).

Luo et al. (89) found that anti-EPOR antibodies in SLE patients were associated with more severe anemia, higher disease activity, augmented anti-dsDNA antibody levels, and lower C3 (increased complement consumption, a sign of disease activity). Notably, Hara et al. specifically looked at 46 patients with biopsy-proven lupus nephritis and detected anti-EPOR antibodies in 18 patients. Those with anti-EPOR antibodies had significantly higher SLE disease activity and more severe anemia, suggesting that anti-EPOR antibodies have inhibitory function. Although these groups shared no differences in anti-dsDNA antibodies, complement levels, or renal function at time of biopsy, those with anti-EPOR antibodies had a higher disease activity index, and the presence of anti-EPOR antibodies was an independent risk factor for CKD progression (90).

Overall, anti-EPO and anti-EPOR antibodies correlate with SLE disease severity and may be associated with poor kidney prognosis, providing associative evidence that, by inhibiting EPOR immune modulatory effects, they may also fuel the autoimmune response.

### EPO's Effects in Murine SLE Models

Different murine models have been developed to investigate pathogenic mechanisms of SLE and to identify potential new targets for therapy (91). While spontaneous models of lupus are principally used to study the genetic susceptibility to the disease, induced models help in defining the role of environmental factors in lupus pathogenesis and identifying mechanisms responsible for the onset and progression of disease. MRL/lpr mice, a spontaneous model of SLE, are characterized by a mutation in Fas gene and develop severe lymphoproliferative disease with lymphadenopathy, splenomegaly, proteinuric nephropathy and skin lesions (92). This strain also shows behavioral abnormalities and cerebritis that resemble neuropsychiatric involvement in SLE (93).

In 2018, Zhang et al. showed that MRL/lpr mice that received EPO for 10 weeks had less urinary protein, lower serum anti-dsDNA antibody levels, lower renal histopathologic scores with less IgG/C3 deposition in glomeruli, and decreased cytokine levels in the kidneys compared to controls. They also found that mice treated with EPO had fewer Th1 and Th17 cells and more Th2 and Treg cells (76).

Another study by Huang et al. (82) found that administration of EPO-derived helix-B peptide (ARA290) to MRL/lpr mice significantly decreased serum levels of antinuclear antibodies (ANA), anti-dsDNA antibodies, creatinine, cytokine levels (IL-6, MCP-1, TNF-α), renal deposition of IgG, and quantity of apoptotic cells in the kidney. Similar results were found in pristane-induced SLE mice. Importantly, these results were obtained without significant changes in erythropoiesis (82).

Mice that lacked EPOR selectively on macrophages developed lupus-like symptoms. At 55 weeks of age, the mice had significantly increased anti-dsDNA, antinuclear, and anti-Smith antibodies, pathologic evidence of increased glomerular deposition of IgG, IgA, and C3, and increased glomerular size, cellularity and infiltration of immune cells compared to controls. They also developed higher proteinuria and serum creatinine and blood urea nitrogen (BUN) concentrations, along with increased IL-6, TNF-α, IFN-α, and IFN-β levels, while TGF-β decreased, suggesting that EPO/EPOR signaling in macrophages is key to maintaining self-tolerance (74).

Furthermore, in pristane-induced lupus-like murine model, EPO therapy increased phagocytosis of apoptotic cells by macrophages and correspondingly decreased accumulation of dying cells. These EPO-treated mice had decreased serum concentrations of anti-dsDNA antibodies, and of IL-6, MCP-1, and TNF-α levels. They also showed decreased glomerular IgG deposition and improved renal function, as indicated by decreased urinary albumin and serum creatinine (74). Mechanistically, these data have been linked to the S1P-EPO-PPARγ pathway in macrophages that is crucial for apoptotic cell phagocytosis (74).

As demonstrated by these studies, EPO treatment reduced disease severity in both pristane-induced and spontaneous MLR/lpr lupus models. More recently, it has been shown that these effects are linked to a direct action of EPO on T cells (78). In these models of lupus nephritis, in which *Epo* gene expression is reduced, EPO treatment prevents Th17 cell induction and increases the Treg/Th17 and Th2/Th1 cell ratio. In pristane-induced lupus nephritis, EPO deficiency selectively on CD4+ T cell resulted in increased susceptibility to the disease (more proteinuria and severe renal involvement) and conferred resistance to the inhibitory effects of EPO on Th17 cell induction (78).

## Is EPOR a Target for Future Immune-Modulating Treatments for SLE?

Erythropoiesis-stimulating agents are already currently used in patients with lupus nephritis for CKD-associated anemia. One cross-sectional study of 12,533 adult patients with ESKD secondary to lupus nephritis found that 4,288 (34%) were receiving ESA therapy at the time of renal replacement therapy (RRT) initiation (94). However, no study has assessed the effect of EPO on renal outcomes in lupus nephritis in humans, including in earlier stages of active disease prior to progression to ESKD.

EPO has immunomodulatory properties that target several pathophysiological mechanisms of SLE. Specifically, EPO has been shown to attenuate proinflammatory cytokine levels, enhance apoptosis and cell clearance, and decrease proliferation of Tconv while promoting Treg induction. Given this background and the EPO-associated positive effects on disease severity in murine models of SLE, EPO may warrant further evaluation in clinical studies including SLE patients.

Notably, EPO administration carries the risk of thrombosis or stroke, especially in patients with a pro-thrombotic disease, like those with SLE. This highlights the potential utility of newer non-hematopoietic EPO-derivatives including carbamylated EPO or ARA290. Although some studies have demonstrated improved lupus nephritis disease activity in mouse models receiving ARA290 (82), others have found inconsistencies between EPO's and ARA290's effects, possibly highlighting the importance of both EPORs in disease pathophysiology (75, 79). Additional studies are needed to clarify the immunomodulating effects of these derivatives and their therapeutic role in SLE.

## Author Contributions

ME wrote manuscript the initial draft. CC participated in the manuscript writing. PC supervised the manuscript. All authors contributed to the article and approved the submitted version.

## Conflict of Interest

The authors declare that the research was conducted in the absence of any commercial or financial relationships that could be construed as a potential conflict of interest.
